# Barriers in Access to Pharmaceutical Care in Greece: The Case Study of the Out-of-Hospital Management of Patients With Acute Asthma

**DOI:** 10.3389/fpubh.2018.00199

**Published:** 2018-07-17

**Authors:** Olympia Konstantakopoulou, Daphne Kaitelidou, Charalampos Economou, Giorgos Charalambous

**Affiliations:** ^1^Department of Nursing, Center for Health Services Management and Evaluation, National and Kapodistrian University of Athens, Athens, Greece; ^2^Panteion University of Social and Political Sciences, Athens, Greece; ^3^Emergency Department, Hippokration General Hospital, Athens, Greece

**Keywords:** barriers in access, pharmaceutical care, acute asthma management, economic crisis, Greece

## Abstract

**Aim:** The objectives of this study were to evaluate the effectiveness of information and administrative assistance regarding patient adherence to asthma guidelines and investigate the nature of the barriers in access to pharmaceutical care in Greece based on the case study of the out-of-hospital management of patients with acute asthma.

**Materials and Methods:** The sample of the study consisted of 100 patients with acute asthma who visited the Emergency Department of a General Hospital of Athens. A comparative cross-sectional study using convenience sampling was conducted during October 2014 and June 2015 regarding the adherence to the follow up.

**Results:** Patients who complied with the follow up visit constituted 61% of the total sample (82% of the patients from the intervention group and 40% from the control group) and for those for whom the follow up visit had been scheduled by the researchers had been compliant with the physician's instructions more often than patients for whom the follow up had not been scheduled by the researchers (OR = 8.2, 95%CI = 2.9–23.2). Patients with increased hospitalization days during the previous year and who did not consume the appropriate medication prescribed for asthma due to lack of a prescription, visited the ED more frequently than the rest of the patients (OR = 271.47, 95%CI = 14.53–5070.8). More than one out of three patients (36.4%) reported that they had not bought their asthma medications because they had no prescription while almost one out of five stated that they had purchased their medications but had used them with savings in doses. Patients who had not taken their asthma medication due to lack of prescription, visited more than once the ED, resulting in non-admission, when compared to patients who had a prescription for their medications. (OR = 3.5, 95%CI = 1.3–9.3).

**Discussion–Conclusions:** Out-of-hospital management of asthma in Greece presents significant gaps and shortcomings, mainly due to important unresolved issues regarding availability, accessibility and use of services. The findings of the present study confirm the cause-effect relationship between ineffective out-of-hospital management of asthma and the increase in the frequency of the use of hospital emergency departments, resulting in an increase in health costs.

## Introduction

Greece is currently undergoing the ninth consecutive year of continuous and profound economic crisis. During these years, the country's GDP fell by 24.8% (from €232.69 billion in 2008 to €174.99 billion in 2016 (1), with the health sector being one of the sectors most affected, much like the neighboring Balkan and Eastern European countries, whose health systems have suffered from the effects of the global recession as well (2). In Greece, the total health expenditure decreased by 35.1% between 2009 and 2016, while public health expenditure declined even further over the same period (44.8%) with mainly horizontal cuts (3). The use of public health services has increased as visits to hospital outpatient clinics have increased more than 2.5% between 2011 and 2013 and admissions have shown a 9% increase between 2010 and 2013 (4).

However, the health system's response to the impact of the crisis in the health sector indicates no integral strategic planning. As the results of a recent study indicate, although the Greek health care system was characterized as inequitable in access and coverage for health even before crisis, the economic crisis and the policies implemented exacerbated existing issues regarding availability, accessibility, acceptability, contact and effective health coverage of the Greek population (5). The issues regarding the uncoordinated access to health services, the system's responsiveness and the increases in informal payments are also met in the Balcan and SSE countries, along with whom Greece is showing common health care performance outcomes and corresponding morbidity (6).

Pharmaceuticals are an area that received special attention in the Structural Adjustment Program (SAP) implemented in Greece after 2010 (2), and a hard ceiling was set for 2012 and subsequent years. According to the SAP, pharmaceutical expenditure should not exceed €2.44 billion in 2013 and €2 billion in 2014, while for the next 3 years it should not exceed €1.94 billion, thus setting a tight upper limit. If the limits were to be exceeded, clawbacks from producers (pharmaceutical companies) would be used to balance the budget. An estimated fall of 39.4% (€2.7 billion) in total (outpatient) pharmaceutical expenditure occurred (from 2009 to 2014), mostly to the benefit of the social health insurance funds which largely fund this expenditure. Between 2010 and 2015, public pharmaceutical expenditure has fallen by 58.8%, reaching the amount of €1.97 billion in 2015 (3).

Given the fact that the Greek health system has been in operation for many years with under-funding of vital sectors but also with insufficient organization, fragmented care and serious primary health care deficits, there are serious doubts about its ability to cope with increased morbidity needs in the coming years. In order to ensure the health system's viability, emphasis must be placed on strategies for optimal use of resources, which undoubtedly should include strengthening primary health care by providing comprehensive and effective out-of-hospital management of chronic diseases such as asthma.

## Out of pocket payments for pharmaceutical care

Greece has always been characterized as quite a “privatized” system, with high out of pocket payments particularly due to public health sector's under-financing. Private expenditure increased as a percentage of total health expenditure during the economic crisis (from 30.5% of THE in 2009 to 39.11% of THE in 2014), compared with a falling trend between 2005 and 2009, bringing Greece to the first position regarding private spending among the EU-15 countries, for which the mean private expenditure has been estimated to 23.6% of THE for 2014. Out-of-pocket payments, representing 35.47% of the total health expenditure (more than 90% of private health expenditure) increased as a percentage of total health expenditure from 28.4% in 2009 to 35.47% in 2014 (3). The figure depicts formal cost-sharing arrangements, direct payments and informal payments, with the latter two representing by far the highest proportion of out-of-pocket payments.

Ambulatory care historically relied to the household budget with more than 60% of the total expenditure on ambulatory services being covered through direct payments in 2014. The lack of planning and coordination, the absence of a well-organized GP-based integrated primary health care system along with the limited capacity of the public PHC sector was some of the reasons fuelling the high direct payments on that sector (7). Although user charges for ESY services are considered to be low, the user charges on pharmaceuticals represent a significant source of funding of the health care system, as the co-payments vary from 0 to 25%, depending on the severity, the chronic nature of the disease and the patients' income.

More specifically, the rate of co-insurance for a drug prescription is almost uniform for all insurance funds at 25%, with the exception of medicines for cancer, diabetes mellitus, psychosis, epilepsy, hemophilia, nanism, renal insufficiency, multiple sclerosis, paraplegia, quadriplegia, and immune system deficiency, for which there is no co-insurance. A co-insurance rate of 10% applies to medicines for low-income pensioners and the following diseases: Parkinson's disease, insipidus diabetes, chronic pulmonary cardiac disease, collagens, osteoporosis, myopathy, inocystic disease, coronary heart disease, tuberculosis and asthma (7).

Since 2011, increases in co-payments for medicines for specific diseases were introduced. Indicatively, a user charge of 10% was introduced for Alzheimer's disease, dementia, epilepsy, angiopathy, Buerger's disease, diabetes type 2, Charcot's disease (there were no user charges prior for these diseases) while for some other diseases (e.g., Coronary heart disease, hyperlipidemia, rheumatoid arthritis, psoriatic arthritis, etc.) the user charges were increased from 10 to 25% (7).

In general, average cost-sharing for pharmaceuticals rose from 13.3% in 2012 to 18% in 2013. Interestingly, only 8% of prescribed drugs (packets) were provided with 0% co-payment in 2013 compared with 13% in 2012 (8). Apart from co-insurance rates, an additional user charge for prescription medicines being the difference between actual and reference prices reimbursed by sickness funds and an extra co-payment fee of €1 for each prescription issued under the national health service (both in primary care and inpatient settings) has been established since 2014. Some exemptions were imposed regarding the €1 per prescription as it doesn't apply for welfare beneficiaries, the uninsured and those belonging to vulnerable groups from August 1st, 2016 and onwards. Additionally, an upper limit was set in 2014 regarding the difference between the actual price and reference price at 20 euro. The uninsured population, the poor and some other vulnerable groups were exempted from the co-payment.

Regarding the poor access to pharmaceuticals, due to the fact that in recent years private pharmaceutical expenditure has steeply increased (and thus burdening the patients as it has been substituting the cutbacks in the public pharmaceutical expenditure) and the lack in research related to access of patients with chronic diseases to medication, such as asthmatic patients, in Greece (whom hospitalization could be avoided if out-of-hospital management of their disease could be improved) led the authors to focus on investigating the respective context and highlight, if proved, the necessity for the chronically ill to comply with their physicians guidelines and for the policy makers in the primary health care sector to support and introduce institutional changes toward improving out-of-hospital management of chronic diseases.

The objectives of this study were to evaluate the effectiveness of information and administrative assistance regarding patient adherence to asthma guidelines (9) and investigate the nature of the barriers in access to pharmaceutical care in Greece based on the case study of the out-of-hospital management of patients with acute asthma.

## The case of patients with acute asthma

### Methodology and design

#### Sample

The sample of the study consisted of 100 patients with acute asthma who visited the Emergency Department (ED) of a General Hospital of Athens. A comparative cross-sectional study was conducted during October 2014 and June 2015 regarding patients' compliance to the follow up, using convenience sampling.

Patients who visited the ED were divided into two groups:

1)The intervention group (this group's follow up was scheduled by the researchers): in this group, the follow up visit was organized by the researchers who undertook the appointment planning procedures. The appointment was scheduled according to the doctor's instructions (approximately 30 days after the visit to the ED) and a reminder (sms) was sent two (2) days before the scheduled date of the follow up visit. Also, during their visit to the ED, they were provided with an information booklet on the factors that cause asthma symptoms and the importance of compliance with treatment, consistency in treatment and communication with the physician.

2)The control group (this group's follow up was not scheduled by the researchers): in this group, no intervention was made by the researchers regarding the arrangement of their follow up; their compliance to the doctor's instructions and attending to the follow up visit was checked by phone.

#### Measurement tools

The questionnaire developed by the researchers included items on access to medication, use of services and socio-demographics. The independent variables of the study were the demographic (and other) characteristics (related to the use of health services, the expenditure they spent on asthma management, the effects of asthma at work and the reasons for not receiving medical care) while the dependent variables (binary) were (a) patients' compliance to follow up or not, (b) visit to ED (due to asthma attacks) followed by admission and (c) visit to ED (>1 times), due to asthma attacks, followed by non-admission.

#### Statistical analysis

Categorical variables are presented as absolute (n) and relative (%) frequencies. The normality assumption was evaluated using the Kolmogorov-Smirnov criterion (*p* > 0.05 for all variables), histograms and normal probability plots. Bivariate analyses were conducted and included Pearson's X^2^ test (Fischer's exact test) and X^2^ test for trend to determine associations between categorical variables and student's *t*-test and Mann-Whitney test and to investigate group differences within continuous variables. Also, multivariate logistic regression was performed (the independent variable which were included in the regression models were those which were significant at the level of 0.2 (*p* < 0.2) in the bivariate analysis) and its results were presented by using the odds ratios, the 95% CIs and the corresponding *p*-values. A two-sided *p*-value of 0.05 was considered statistically significant. The Statistical Package for Social Sciences (IBM SPSS) program, version 20.0, was used for statistical analysis.

#### Ethical issues

None of the authors have any kind of financial, consultant, institutional or other relationships that might lead to bias or a conflict of interest in the manuscript. All procedures performed in the study were in accordance with the ethical standards of the institutional research committee and approval of the research protocol was ensured by the Scientific Committees of the public hospital. Finally, informed consent was obtained from all individual participants included in the study.

### Results

The patients' mean age was 48.17 years (*SD* = 18.03) and 68% were female. Almost one out of three (31%) were primary school graduates, 52% were high school graduates and 17% were graduates of higher/upper secondary education. Fifty-one percent were working, 29% were unemployed and 20% were retired. The average working time in hours per week was 20.98 h (*SD* = 23.21) and 88% had insurance coverage (Table 1).

**Table 1:** Sample characteristics (demographics, use of health services, use of medication and absenteeism from work/loss of productivity).

**Characteristic**	***N* (%)**
**Gender**	
Man	68 (68.0)
Woman	32 (32.0)
**Age (in years)**^a^	48.17 (18.04)
**Working hours per week**^a^	20.98 (23.21)
**Educational level**	
Primary school	31 (31.0)
High school	52 (52.0)
Higher/upper secondary education	17 (17.0)
**Insurance status**	
Insured	88 (88.0)
Not insured	12 (12.0)
**Working status**	
Employed	51 (51.0)
Unemployed	29 (29.0)
Retired	20 (20.0)
**Frequency of visiting the doctor due to asthma attacks (over the preceding 12 months)**^a^	2.78 (2.86)
**Visits to the doctor due to asthma attacks**	
Never	10 (10.0)
Once or Twice	51 (51.0)
3–8 times	36 (36.0)
10–12 times	2 (2.0)
≥20 times	1 (1.0)
**Visits to the ED due to asthma attacks not followed by hospital admission (over the preceding 12 months)**	
Once	63 (63.0)
At least twice	37 (37.0)
**Visits to the ED due to asthma attacks followed by hospital admission (over the preceding 12 months)**	
Never	68 (68.0)
At least once	32 (32.0)
**Frequency of not visiting the doctor due to underestimating the significance of their health problem**	
Never	57 (57.0)
At least once	43 (43.0)
**Prescription for asthma medication each month (over the preceding 12 months)**	
No	84 (84.0)
Yes	16 (16.0)
**Not purchasing asthma medication due to lack of prescription**	
No	63 (63.6)
Yes	36 (36.4)
**Additional obstacles in getting a prescription due to the completion of the prescription limit (over the preceding 12 months)**	
No	76 (80.0)
Yes	19 (20.0)
**Days of absence from work due to asthma (over the preceding 12 months)**	
0 days	52 (52.0)
1–7 days	22 (22.0)
≥8 days	26 (26.0)
**Self-estimated rate of decline in work**^a^	39.5 (32.3)
**Decline in efficiency/performance at work**	
0–20%	40 (40.0)
21–40%	10 (10.0)
41–60%	22 (22.0)
61–80%	26 (26.0)
81–100%	2 (2.0)

a *Mean value (standard deviation)*.

The intervention team and the control group consisted of 50 patients each and the patients who complied with the follow up visit constituted 61% of the total sample size; 82% of the patients from the intervention group and 40% from the control group (Table 2).

**Table 2:** Significant relations between demographic (and other characteristics) and patients' compliance to follow up.

	**Patients' compliance to follow up**, ***n*** **(%)**		***p*-value**
	**No**	**Yes**	
**Insurance coverage status**			0.01^a^
Uninsured	9 (75.0)	3 (25.0)	
Insured	30 (34.1)	58 (65.9)	
**Visiting the ED due to asthma attacks with no subsequent admission to hospital**			0.05^b^
1 time	20 (31.7)	43 (68.3)	
More than 1 time	19 (51.4)	18 (48.6)	
**Patients' Group**			<0.001^c^
Control group	30 (60.0)	20 (40.0)	
Intervention group (scheduled follow up)	9 (18.0)	41 (82.0)	

a X^2^ test.

b *X^2^ test for trend*.

c *Fischer's exact test*.

In fact, patients for whom the follow up visit had been scheduled by the researchers had been compliant with the physician's instructions more often than patients for whom the follow up had not been scheduled by the researchers; the likelihood of compliance to the follow up was 8.22 times higher for patients for whom the follow up visit had been scheduled by the researchers. Also, the insured patients complied with the follow up visit more often than the uninsured ones (OR = 14.63) (Table 3).

**Table 3:** Multivariate logistic regression (Dependent variable: Patients' compliance to follow up).

	**Odds ratio**	**95% CI**	***p*-value**
Patients with scheduled follow up visit in relation to control group's patients	8.22	2.90–23.27	<0.001
Insured patients in relation to uninsured ones	14.63	2.10–101.8	0.007

#### Visits to the doctor and ED

Patients with increased hospitalization days during the previous year and who did not consume the appropriate medication prescribed for asthma due to lack of a prescription, visited the ED more frequently than the rest of the patients. In fact, during the 12 months which preceded the study, patients visited the doctor due to asthma attacks on an average of 2.78 times; in particular, 36% of the asthma patients visited the doctor from three to eight times, 37% visited the ED due to an asthma attack at least twice and 32% visited the ED and were hospitalized at least once (Table 1). Additionally, 43% of the patients had not visited their doctor for at least one time because they did not consider their problem to be of significance and one out of four stated that they had not visited their doctor at least once because they were unable to pay him/her (Table 1). Finally, the likelihood of visiting the ED more than 1 times (due to asthma attacks), followed by non-admission, was 4.22 times higher in patients who did not visit their doctor (although they had a crisis) because they did not consider their problem to be important compared to patients who attributed the necessary importance to their problem (Table 4).

**Table 4:** Multivariate logistic regression (Dependent variable: Visiting the ED, more than once, due to asthma attacks followed by non-admission).

	**Odds ratio**	**95% CI**	***p*-value**
Not visiting the doctor (although having a crisis) because they did not consider their problem to be important	4.22	1.27–13.97	0.019
Non adherent to medication regimens patients in relation to adherent patients	3.52	1.33–9.33	0.011

Patients with uncontrolled/severe asthma, as classified according to asthma hospitalization days in the previous year, experienced frequent attacks and visited the ED more often (OR = 271.47) (Table 5).

**Table 5:** Multivariate logistic regression (Dependent variable: Visiting the ED due to asthma attacks followed by hospital admission).

	**Odds ratio**	**95% CI**	***p*-value**
Days of hospitalization due to asthma in the previous year	271.47	14.53–5070.8	<0.001

#### Medication

A significant percentage of patients with asthma did not consume the medication prescribed either due to lack of a prescription or due to lack of economic means to buy the medication and/or to pay the cost-sharing expenses and a significant number of patients did not adhere to their physician's guidelines about the appropriate dosage in order to save doses and, thus, to extend the duration of the use of the medication. In particular, only 16% of the patients had received a prescription for their asthma medication each month (over the preceding 12 months) (Table 1), while almost 2 out of 5 patients paid a monthly contribution for asthma medications from 21 to 60 Euros. Overall, 35% of the sample population stated that they paid from the family budget for expenses related to their asthma condition from 51 to 100 Euros per month, reporting that the total cost of buying medication (both for asthma and any other illnesses) was very high. More than one out of three patients (36.4%) reported that they had not bought their asthma medications because they had no prescription while almost one out of five stated that they had purchased their medications but had used them with savings in doses (Table 1). Furthermore, 20% of the patients reported additional prescription difficulties for their asthma medications due to the monthly prescription limit (Table 1). Finally, patients who had not taken their asthma medication due to lack of prescription, visited more than once the ED (due to asthma attacks), resulting in non-admission, compared to patients who had a prescription for their medications. In fact, the likelihood of an ED visit more than once (due to asthma attacks), followed by non-admission, was 3.52 times higher in patients who did not take their asthma medications due to lack of prescription compared to patients who had a prescription for their medication (Table 4).

#### Absenteeism from work/loss of productivity

During the 12 months which preceded the study, 22% of the study patients stated that they were absent from their work due to asthma from 1 to 7 days, while the self-estimated mean rate of decline in work, related to asthma was 39.5%; in fact, two out of five patients reported that their efficiency/performance at work dropped from 0 to 20% and 32% from 21 to 60% (Table 1).

## Discussion–conclusions

This case study has clearly brought to light that when out-of-hospital management of asthma is unsuccessful and ineffective, the incidence and cost of hospitalizations, visits to emergency departments and other related forms of care are increasing (10, 11).

Asthma is one of the most prevalent chronic respiratory disease worldwide along with COPD (12) and, according to the OECD and the analysis of the key indicators for health and health systems in 34 member countries (13), the indicator for asthma (defined as the number of patients who are discharged after hospital treatment per 100,000 population) is one of the most important indicators in terms of running a country's PHC. The rationale for selecting this indicator was that a large proportion of these hospitalizations could have been avoided if the management of asthma patients was timely and successful in primary health care structures as the prevention of possible exacerbations associated with out-of-hospital management would be ensured. Also, medication-related factors such as cost of medication (14, 15) and others such as poor supervision/training and complacency (16) are usually associated with non-adherence to asthma therapy.

In Greece, and especially at the current juncture with the economic crisis to deepen and the health resources to decrease continuously, avoidable hospitalizations through effective out-of-hospital management of chronic diseases should be a basic objective of the health system. This could be a means toward rationalizing health costs and saving resources in order to meet the growing demand of public health services. However, according to the findings of our study, a very significant percentage of patients with asthma did not comply with the prescription medication either because they did not have a prescription or because they had no money to buy medicines and/or to pay sharing costs or because it was not considered to be necessary. A very significant percentage purchased the medication; nevertheless, they were not used in the recommended dosages in order to make savings and thus, extend the duration of the use of the medicine. Similar findings have been found in other studies conducted to assess the impact of the economic crisis on access and use of health services by chronic patients, not only in Greece but also in other European countries (17). For example, in a nationwide survey conducted by the National School of Public Health (18) on the economic crisis and its consequences for patients with chronic diseases, it is noted, inter alia, that 10% of the chronic patients reported that they were unable to receive their medication due to a decrease in their income. Finally, 40% of participants did not comply with their follow up visit in order to redefine their therapeutic plan, which is essential for the effective management of asthma However, the intervention group patients were observed intensively and reminded of the approaching visits something that may improve adherence to follow-up and leaving room for a bias; but also highlighting the importance of these effective administrative interventions when managing chronic patients.

Patient accessibility problems in health services also appear to be triggered by the set limits for the monthly allowed prescription rates per doctor, as 20% of the patients stated that they had to visit at least two physicians for an asthma medication prescription (due to the monthly prescription limit). Also one out of four patients reported that they did not visit their doctor because they were not able to pay him/her (this study was conducted before the nationally initiated reform i.e., legislation to provide comprehensive health insurance coverage to the unemployed and vulnerable groups in 2016).

From the above, it is clear that out-of-hospital management of asthma presents significant gaps and shortcomings either due to important unresolved issues with regards to availability, accessibility and use of services, either due to the fact that health policies have not preceded so as to strengthen these care dimensions. The findings of the present study confirm the cause-effect relationship between ineffective out-of-hospital management of asthma (not using medication at all or properly, disregarding the significance of the disease, etc.) and the increase in the frequency of the use of the EDs' health services, resulting in an increase in the health costs by the use of the EDs. The improper out-of-hospital management not only causes congestion in the functioning of the EDs, but also it contributes to increases in costs, compared to the cost of outpatient visits in order to monitor the course and management of the disease. It is therefore obvious that effective out-of-hospital management of the disease contributes to reducing the use of health services, resulting in a reduction in the cost of hospitalization and the use of EDs' services.

## Author contributions

All authors listed have made a substantial, direct and intellectual contribution to the work, and approved it for publication. In particular, OK helped supervise the study, conducted the field research, performed the statistical analysis and wrote the manuscript with support from the co-authors. DK conceived the original idea and methodological design, supervised the study and the findings of this work, discussed the results and contributed to the final manuscript. CE discussed the results and contributed to the final manuscript. GC discussed the results and contributed to the final manuscript.

### Conflict of interest statement

The authors declare that the research was conducted in the absence of any commercial or financial relationships that could be construed as a potential conflict of interest.
